# EXAMINING CUSTODIAL GRANDMOTHER’S PARENTING PRACTICES BY MEANS OF A THREE-STEP LATENT PROFILE ANALYSIS

**DOI:** 10.1093/geroni/igae098.1486

**Published:** 2024-12-31

**Authors:** Minzhi Ye, Gregory Smith

**Affiliations:** University of Texas Rio Grande Valley, Edinburg, Texas, United States; Kent State University, Kent, Ohio, United States

## Abstract

Although parenting is the central responsibility of custodial grandparents, research on the parenting practices of these family caregivers remains scarce. With various measures of parental nurturance and discipline as objective indicators, we used Three-Step Latent Profile Analysis (Mplus 8.1) to (1) identify distinct custodial grandmother (CGM) parenting profiles among a nationwide sample of 733 individuals and (2) examine how these profiles were related to grandchildren’s (GC) psychological difficulties, CGM mental health, CGM coping behaviors, and key contextual factors. Four parenting profiles emerged: Permissive (35%; n = 259), Authoritative (7%; n= 53), Optimal (49%; n = 357), and Inconsistent (9%, n=64). These profiles differed considerably in terms of how each was related to CGM depressive and anxiety symptoms as well as to GC internalizing and externalizing difficulties. For example, in Permissive CGM, both their anxiety and depression symptoms were positively related to GC internalizing and externalizing difficulties. On the other hand, among Authoritative CGM, their depressive symptoms were negatively related to GC internalizing, while their anxiety symptoms were positively related to CG internalizing. In addition, multinomial contrasts with Optimal CGM as the referent showed that “Permissive” CGM reported less problem-focused coping, more family conflict, and younger CGM with older GC. “Authoritative” CGM reported higher depressive symptoms, less support, and increased family conflict. Finally, Inconsistent CGM reported less problem-focused coping, heightened family conflict, and older GC. These findings shed light on the relationships between CGM parenting profiles, well-being, coping behaviors, and GC outcomes which informs future interventions. [Funded by NIH R01- MH-66851-02]

